# Completion lower lobectomy after basal segmentectomy for pulmonary sclerosing pneumocytoma with lymph node metastasis

**DOI:** 10.1093/jscr/rjab492

**Published:** 2021-11-11

**Authors:** Hiroaki Komatsu, Nobuhiro Izumi, Takuma Tsukioka, Hidetoshi Inoue, Ryuichi Ito, Noritoshi Nishiyama

**Affiliations:** Department of Thoracic Surgery, Osaka City University Hospital, Osaka, Japan; Department of Thoracic Surgery, Osaka City University Hospital, Osaka, Japan; Department of Thoracic Surgery, Osaka City University Hospital, Osaka, Japan; Department of Thoracic Surgery, Osaka City University Hospital, Osaka, Japan; Department of Thoracic Surgery, Osaka City University Hospital, Osaka, Japan; Department of Thoracic Surgery, Osaka City University Hospital, Osaka, Japan

## Abstract

A 20-year-old man was referred to our hospital because of a tumor in his left lung. Chest computed tomography showed a 35-mm nodule in the left lower lung lobe. Bronchoscopic examination and cryobiopsy were performed, which revealed suspicion for sclerosing pneumocytoma. We performed basal segmentectomy, and intraoperative-frozen pathological examination revealed no metastases in the segmental lymph nodes. However, the postoperative pathological diagnosis revealed metastasis in these nodes. We performed additional resection of segment 6 (completion lower lobectomy) and hilar and mediastinal lymph node dissection 2 weeks after the first surgery. The postoperative course was favorable, and the patient was discharged 13 days after the second surgery. Pathological examination of the additional resected specimens revealed lymph node metastases in the interlobar and mediastinal lymph nodes. Pulmonary sclerosing pneumocytoma with lymph node metastasis is extremely rare, and its prognosis is unclear. Recurrence has been reported rarely, and long-term follow-up is required.

## INTRODUCTION

Pulmonary sclerosing pneumocytoma is a rare benign or low-grade malignant tumor of the lung, and cases with lymph node metastasis are extremely rare [1–9]. We herein report a patient with pulmonary sclerosing pneumocytoma with lymph node metastasis who underwent completion lower lobectomy after basal segmentectomy.

## CASE REPORT

**Figure 1:**
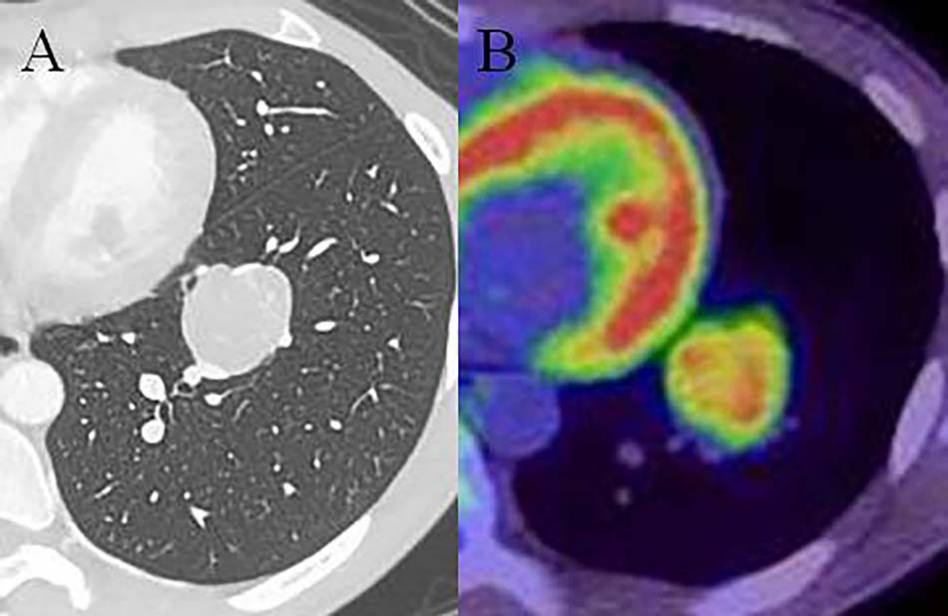
Chest computed tomography (CT) showing a 35-mm nodule in the lower lobe of the left lung (**A**). Fluorodeoxyglucose-positron emission tomography (FDG-PET) showing high accumulation in the nodule (SUV_max_: 5.9; **B**).

**Figure 2:**
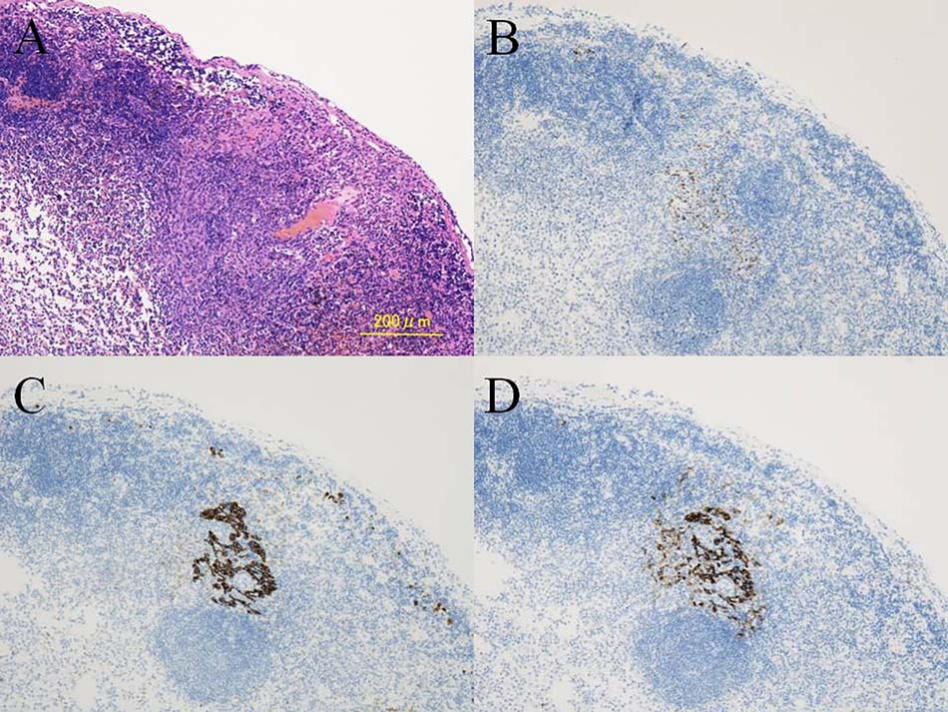
Histological findings showing the metastatic tumor in the mediastinal lymph nodes (**A**). Immunohistochemistry demonstrating positive staining for TTF-1 (**B**), AE1/AE3 (**C**), and EMA (**D**) in the metastatic tumor cells.

A 20-year-old man was referred to our hospital because of a tumor in his left lung that was discovered on chest radiographs. Chest computed tomography (CT) showed a 35-mm nodule in the lower lobe of the left lung (Fig. 1a). Fluorodeoxyglucose-positron emission tomography (FDG-PET) showed high accumulation in the nodule (maximum standard uptake value (SUV_max_): 5.9; Fig. 1b). There was no accumulation in the hilar and mediastinal lymph nodes. Bronchoscopic examination and cryobiopsy were performed, which indicated suspicious for sclerosing pneumocytoma. To confirm the diagnosis and for treatment, we performed video-assisted thoracoscopic surgery (VATS). Intraoperative frozen pathological examination revealed that there were no metastases in the segmental lymph nodes (No. 13). Then, we performed basal segmentectomy considering curability and the preservation of pulmonary function. The operating time was 154 min and blood loss was 30 ml. However, the postoperative pathological diagnosis revealed lymph node metastasis in the segmental lymph nodes. Considering the possibility of remaining lymph node metastases, we performed additional resection of segment 6 (completion lower lobectomy) and hilar and mediastinal lymph node dissection by VATS, 2 weeks after the first surgery. The operating time was 266 min and blood loss was 200 ml. Hilar adhesions were moderate, and these were carefully dissected to avoid injuring the pulmonary artery. Then, the pulmonary artery was safely cut using a stapler. The bronchus of the left lower lobe was cut at the root using a stapler, followed by radical hilar lymph node dissection. The patient’s postoperative course was favorable, and he was discharged 13 days after the second surgery. Pathological examination of the second resected specimen revealed lymph node metastases in the interlobar (No. 11) and mediastinal lymph nodes (No. 4 L, 7 lymph nodes; Fig. 2). Six months after the surgery, he was alive without no recurrence or metastasis.

## DISCUSSION

Reviewing studies of pulmonary sclerosing pneumocytoma, there were 25 cases of pulmonary sclerosing pneumocytoma with lymph node metastases [1–9]. Curative resection is the best treatment; however the standard surgical procedure has not been established. Most patients with lymph node metastases underwent lobectomy with lymph node dissection according to the preoperative or intraoperative diagnosis of suspicious for lung cancer. Among the 25 patients, only two patients underwent segmentectomy [8, 9]. Although some authors reported that lymph node metastases did not affect the prognosis [2, 3], the mean follow-up period in previous reports was only 27 months [1]. In addition, it is uncertain whether remaining tumor in the lymph nodes will grow. Thus, the long-term outcome of patients undergoing segmentectomy without lymph node dissection is unclear. Therefore, we performed additional resection of segment 6 (completion lobectomy) and hilar and mediastinal lymph node dissection, in our patient.

It is difficult to make a correct diagnosis of sclerosing pneumocytoma, either preoperatively or intraoperatively [1, 2]. In fact, intraoperative frozen pathological examination did not identify lymph node metastasis, in the present patient. Postoperative histological and immunohistochemical examinations are the main methods of diagnosing sclerosing pneumocytoma [2]. In patients undergoing resection for lung cancer, discrepancies between clinical, surgical and pathological lymph node diagnoses cannot be avoided, and pathological lymph node metastasis in permanent sections may be seen in some patients [10, 11]. Completion lobectomy is recommended in these patients, although the significance of additional completion lobectomy remains unclear for patients with unexpected lymph node involvement [10, 11]. In the present patient, additional curative resection was performed according to the standard therapeutic strategy for lung cancer.

Completion lobectomy after segmentectomy is extremely difficult because of severe adhesions around hilar structures [11, 12]. Completion lobectomy is the treatment of choice for patients with local recurrence, a second primary lung cancer, or metastatic lung cancer after segmentectomy for lung cancer. However, completion lobectomy may become more difficult to perform more than 5 weeks after segmentectomy owing to severe adhesions [11]. Therefore, we performed completion lobectomy as early as possible after the postoperative diagnosis of lymph node metastasis. Two weeks after the first surgery, hilar adhesions were moderate, and we were able to divide and cut the pulmonary artery safely, using VATS.

Previously, sclerosing pneumocytoma was considered benign; however, cases with bone metastasis or pleural spread have been reported [13, 14]. Among the 25 cases with lymph node metastases, one patient had recurrence in the opposite lung, and the second resection was performed [15]. Some researchers believe that sclerosing pneumocytoma has malignant potential [2, 4], whereas there have been no deaths reported owing to this disease, to date. Survival data are available for a very limited number of patients, and long-term surveillance of patients with sclerosing pneumocytoma with lymph node metastasis is required.

In conclusion, we successfully performed complete lower lobectomy 2 weeks after basal segmentectomy in a patient with pulmonary sclerosing pneumocytoma with lymph node metastasis. Recurrence after surgery has been reported rarely, and long-term follow-up is required.
